# Step down Vascular Calcification Analysis using State-of-the-Art Nanoanalysis Techniques

**DOI:** 10.1038/srep23285

**Published:** 2016-03-16

**Authors:** Sven C. Curtze, Marita Kratz, Marian Steinert, Sebastian Vogt

**Affiliations:** 1 Department of Materials Science, Tampere University of Technology, Tampere, Finland; 2 Institute for Experimental Orthopaedics and Biomechanics, Philipps University, Marburg; 3 Heart Surgery, Universitätsklinikum Gießen und Marburg GmbH, Germany

## Abstract

New insights into the architecture and formation mechanisms of calcific lesions down to the nanoscale open a better understanding of atherosclerosis and its pathogenesis. Scanning electron – and atomic force microscope based nano-analytical characterization techniques were adapted to the assessment of an *ex-vivo* calcified coronary artery. Human atherosclerotic tissue and bone tissue reside a typical chemistry of Magnesium and Sodium rich Calcium phosphates, identified as whitlockite and Calcium apatite, respectively. Despite the obvious similarities in both chemistry and crystallography, there are also clear differences between calcified vascular tissue and bone such as the highly oriented growth in bone, revealing meso-crystal character, as opposed to the anisotropic character of calcified vascular lesions. While the grain size in vascular calcified plaques is in the range of nanometers, the grain size in bone appears larger. Spherical calcific particles present in both the coronary artery wall and embedded in plaques reveal concentric layers with variations in both organic content and degree of hydration.

Despite a large number of studies on molecular mechanisms^1^,^2^,^3^,^4^,^5^ of the five known forms of vascular calcification^6^,^7^, major parallels are revealed to bone development. The description of bio-apatite plaques’ architecture and microstructural aspects such as crystallography, grain size, crystal orientations and growth directions as well as ultrastructural arrangements remains vague because of two facts: The nanocrystalline nature of the biomineral phase and the intimate association of the mineral phase with macromolecular collagen^8^.

The majority of earlier studies on the crystallography of vascular calcification stem from bulk analysis techniques using X-ray diffraction (XRD) or synchrotron radiation^3^,^4^,^9^,^10^,^11^,^12^,^13^,^14^,^15^,^16^, where the phase is identified from crystallographic lattice plane reflections and grain size is indirectly determined from peak broadening. Other studies on vascular calcification have used transmission electron microscopy (TEM)^5^.

## Results

**Figure 1:**
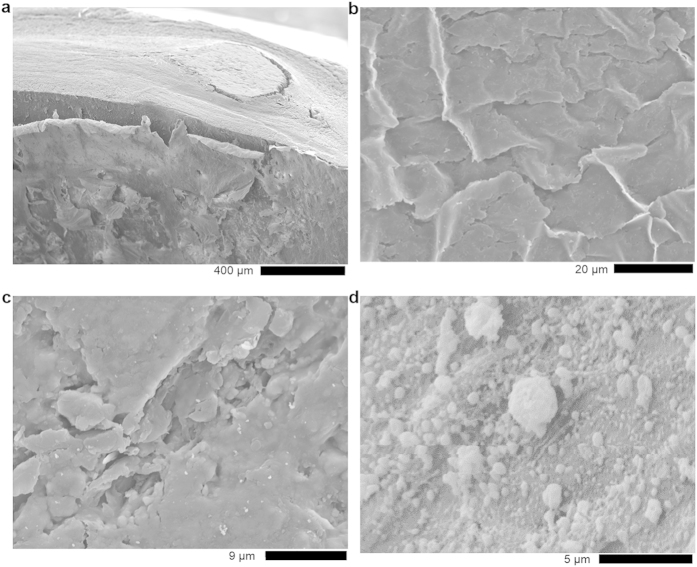
SEM-micrographs of human aortic tissue with calcific plaque. Overview (**a**), inner wall covered with a thin plates (**b**), higher magnification of compact calcified plaque (**c**). Dense spherical particles in different cluster sizes in the inner wall of the coronary artery (**d**).

Figure 1 shows the Scanning Electron Microscope (SEM) -photographs of the human coronary artery, exhibiting compact calcified material embedded between the media and an enormous fibrolayer (Fig. 1a). The wall thickness was determined as 1.1 mm. Parts of the inner wall are covered with thin plates as can be seen in Fig. 1b. An image taken at higher magnification from the compact calcified plaque (Fig. 1c) suggests the presence of spherical particles embedded in material of different composition. A distribution of dense spherical particles in different cluster sizes are found in the inner wall of the coronary artery, shown in Fig. 1d. According to Bertazzo *et al.*^5^, spherical particles represent the initial stages of mineralized structure, closely related to disease initiation, preceding the other known morphological variants forming with progressing disease.

The chemical composition of the calcified lesion and the bone sample obtained by energy dispersive X-ray spectroscopy (EDX) analysis are shown in Table 1. Both materials reveal chemistry typical of magnesium and sodium rich calcium phosphates. The calcium to phosphorus ratio in the calcified vascular lesion is Ca/P ≤ 1.5. Repeated measurements were quite consistent. The standard deviations over a series of six measurements from different sample spots are also shown in Table 1. The Ca/P-ratio suggests a composition close to that of Mg/Na-substituted tricalcium phosphate, commonly referred to under the mineral name whitlockite^8^. The variance in the EDX-results on the trabecular bone sample is somewhat higher, showing Ca/P-ratios varying with sample location between 1.4 and slightly above 1.6, which is close to that of ideal hydroxylapatite (Ca/P-ratio = 1.67). A Ca/P-ratio below 1.5 is indicative of non-apatitic structure^17^,^18^. Inconsistencies in the quantitative results are most likely to be due to material inhomogeneity.

**Figure 2:**
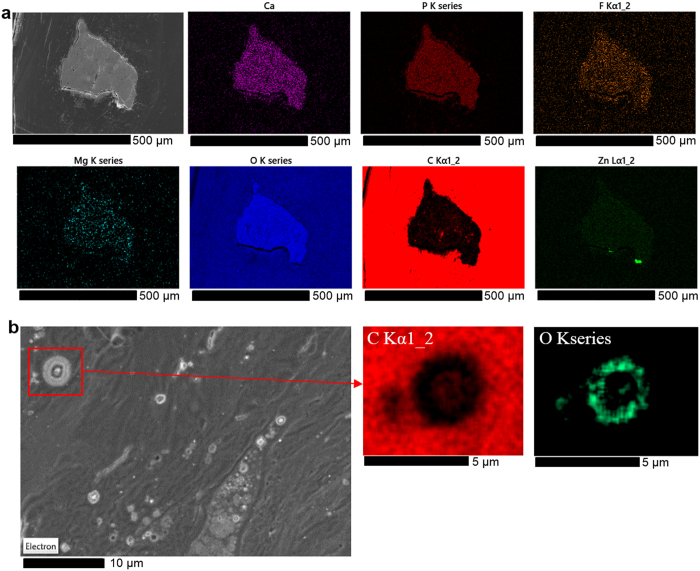
EDX-mappings of aortic plaque. Mapping over the calcified plaque sample (**a**) and an area showing dense spherical particles (**b**) at 5 kV accelerating voltage. Due to the C-Kα-line-Ca-Lα-line overlap, deconvolution of these maps did not work properly. Therefore, the only weakly excited Ca-Kα-line was used instead, showing less intensity, and giving the impression of lower Ca-content, than actually present.

EDX-mappings over the region of interest are shown in Fig. 2. The large calcified lesion is well defined from the surrounding arterial wall. The carbon mapping shows high carbon content in the arterial wall and minor fractions in the large calcified plaque, with organic content distributed inhomogenously. The enormous calcified lesion (<500 μm X 500 μm) contains Mg, Na, P, and O in addition to Ca. Also fluorine was detected in smaller quantities. High zinc concentration was detected at the interface between plaque and arterial wall in two positions. The origin of increased Zn concentration at these spots has not been further investigated yet, but Zn playing an important role in immune response, being associated with white blood cells^19^. Also matrix metalloproteinases (MMP) in osteoclasts carry a Zn^2+^ ion in their active catalytic site, which might possibly be related to the increased occurrence of Zn here. Smaller spherical mineralized foci with the same chemistry as in the large calcified lesion could be detected in the artery wall, as can be clearly seen from the higher magnification micrograph and EDX maps in Fig. 2b. The small spherical particles show concentric multilayers, where lighter and more dense material interchanges. While some of the particles represent a dense core, followed by periodically repeating light and dense material layers, others show a light core, followed by repeatedly interchanging dense and light layers. From the EDX mapping on one of the larger particles (Fig. 2b) one can distinguish high organic content in the innermost light element ring, surrounding the oxygen rich core, while increased oxygen content is detected also towards the outer shell of the sphere, suggesting that the variation in density correlates with organic content and degree of hydration. Diffusion-controlled mechanisms might play a role in the biomineralization process.

**Figure 3:**
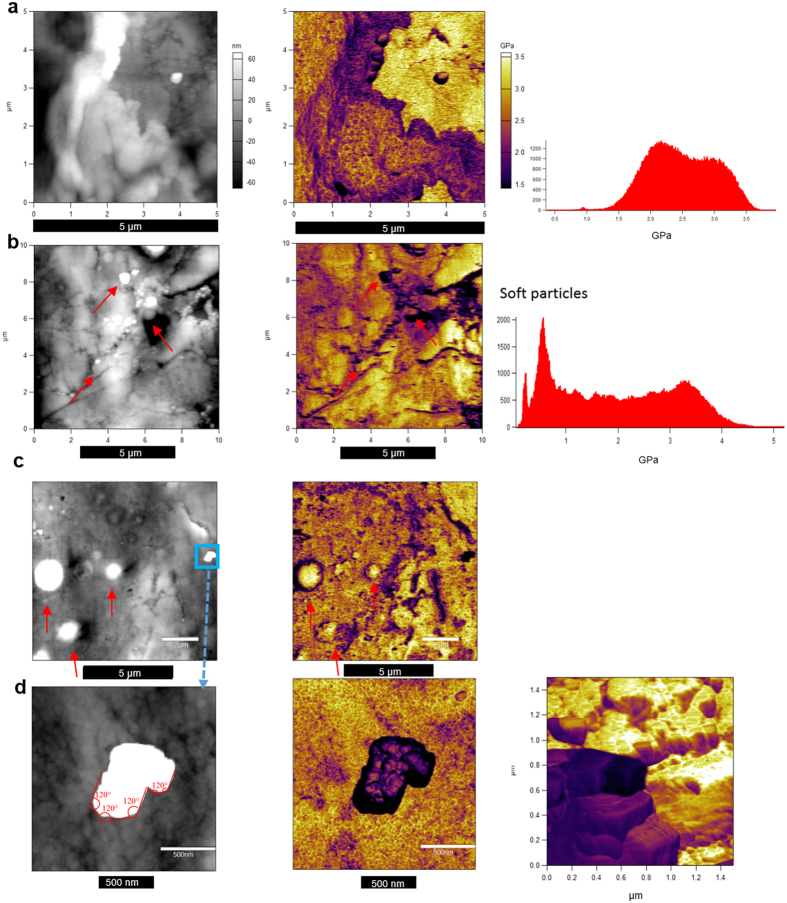
AM–FM measurements on the large calcified plaque. 5 × 5 μm scan area height map and Young’s modulus map indicating two ‘phases’. The diagram shows the frequency distribution of Young’s moduli (**a**). 10 μm^2^ area of interest scan showing presence of soft particles, more or less spherical (**b**). 10 μm^2^ area of interest scan showing presence of spherical particles revealing a stiff core and soft outer shell/transition zone towards the matrix (**c**). Zoom-in on the region highlighted in (**c**), containing a softer particle, including a 3D overlay colour map from AF-MF-Young’s modulus data onto a topography image (**d**).

The amplitude modulation – frequency modulation (AM–FM) measurements over several areas within the large calcified plaque lesion in general reveal a bi-modal distribution of Young’s moduli, with means around 2.2 and 3 GPa (Fig. 3a), probably attributed to variations in organic content. Smaller and larger areas of similar stiffness can be observed, as visible from Fig. 3. From Fig. 3d, a foam-like network structure can be observed showing a colloidal texture, with nanograins of approximately 20 nm in diameter. A very striking observation in this respect is higher stiffness of the nano-grainboundary network than the stiffness within grains. Studies on mineral in nacre^20^ have evidenced a quite similar grain structure with a crystalline grainboundary network of lower stiffness than the inside grains, which was interpreted as organic network. In the present case high organic content along grain boundaries is rather unlikely judging from the high stiffness. Taken together, the organic content does not seem to distribute between the nanograins, but rather seems to distribute inhomogeneously on the micron level.

The nucleation, growth, and coalescence of the nanoparticles/nanograins have a key role in the understanding of the biomineralization process. Different theories have been presented, reviewed by Gower^21^. The quite different character of the analyzed bulky lesions compared to spherical particles is obvious. After sectioning with a focussed ion beam, the finding of highly crystalline hydroxylapatite was reported^5^.

In general, crystals will strive to minimize their Gibbs free energy, where higher crystalline order goes along with a lower energetic state. Therefore, spherical particles are unlikely to transform into the larger biomineralized lesions of lower order, but the different morphological variants rather form alongside, or a sort of mineral cement forms around the spheres subsequently, where spheres might act as nucleation sites. The amorphous precursor pathway (APP), the polymer-induced liquid-precursor (PILP) theory in particular, is an interesting hypothesis to explain the different observed morphological calcification variants, including nanocrystalline lesion as well as the highly crystalline spherical particles, where the observed concentric layers in spheres can be explained by unequal proportioning of organic content due to diffusion-limited exclusion of organics^21^. Also by AM–FM measurements, spherical particles can be observed (Fig. 3b,c) in addition to other morphological particle variants by AM–FM. Larger spheres, approximately 1 μm in diameter show highest stiffness (Young’s modulus up to 3.5 GPa) towards the centre, while outermost layers expose distinctly lower stiffness (Fig. 3c). The Data is quite analogous to the EDX mappings and confirms differences in organic content and hydration. There are many smaller particles (Fig. 3b), many of which are spherical while others deviate by varying distinctness from spherical morphology, softer than the base material with Young’s moduli roughly in the range of 100–700 MPa, i.e., just above that of living cells (in the range of protein crystals such as agglomerated white blood cells or Insulin^22^,^23^,^24^. The particles reveal some pane like straight edges with angles of approximately 120° in-between (Fig. 3d), which could indicate hexagonal or trigonal crystal symmetry. A 3D overlay colour map from Young’s modulus data onto a topography image from such a particle is shown in Fig. 3d, where also lattice fringe resembling morphology can be observed on the surfaces. The nature of these particles and their potential role in the lesion formation is purely speculative at the moment. Studies on biomineralization processes of Calcium Phosphate^13^,^14^,^25^, for instance in bone formation in Zebrafish fin rays^14^, typically involving some sort of amorphous calcium phosphate, e.g., Octacalcium Phosphate, that is gradually converted into fully crystalline phase, describe intermediate phases that closely resemble the observed particles.

**Figure 4:**
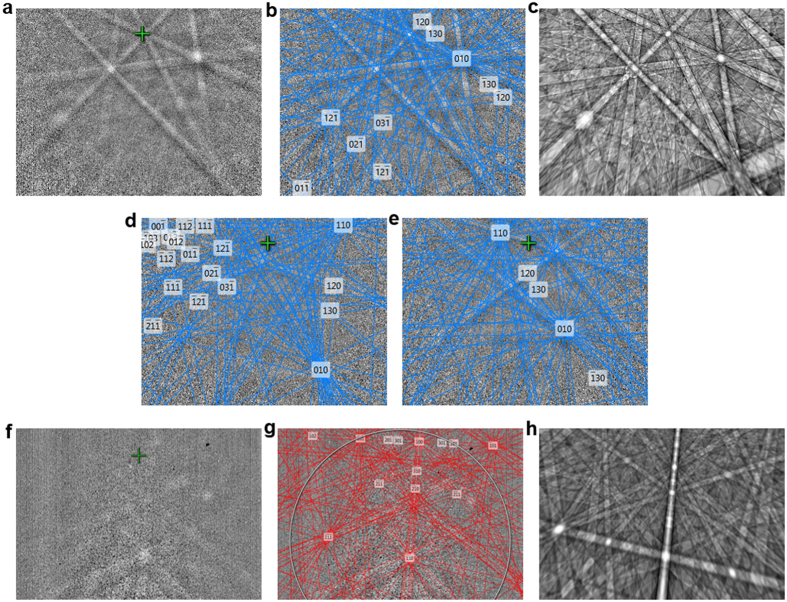
Electron diffraction pattern from the vascular calcification lesion and bone. Kikuchi pattern (EBSP) from the vascular calcification lesion (**a**), solution of the phase after band detection (**b**), dynamic simulation of the pattern (**c**) and further solved measurement points of different orientation (**d**,**e**). Kikuchi pattern from the bone sample as acquired in TKD set-up (**f**), solution of the phase (**g**), and dynamic simulation based on the data obtained from the solution (**h**).

Diffraction pattern were acquired from several positions on the arterial plaque sample which allowed indexing of the present phase as Sodium/Magnesium whitlockite Ca_10_Na(Mg)(PO_4_)_7_ with trigonal-high (−3 m) crystal symmetry, space group 161 (Fig. 4). The nominal composition of the phase (O = 61%, Ca = 22%, P = 15%, Na = 2%) and the Ca/P ratio ≤ 1.5 appears to be an almost perfect match when compared to chemical composition in Table 1, suggesting minor substitutions of Ca^2+^ ions by Na^+^ and Mg^2+^ ions. Figure 4c shows a dynamic simulation of the pattern in Fig. 4a, confirming that the whitlockite lattice can in fact be described by the observed band arrangement and also the intensities of the major reflexes match. The crystal orientation determined at different positions changed, not revealing any preferred orientation of grains (Fig. 4d,e).

The diffraction pattern of the bovine trabecular bone sample and its indexing solution is shown in Fig. 4f,g, whereas pattern quality was low. Due to the changed projection geometry, i.e., transmission Kikuchi diffraction with an almost horizontally aligned sample as compared to the conventional electron backscatter diffraction (EBSD) measurements on the calcified artery sample, the pattern distortion is stronger especially towards the bottom side of the phosphor screen, and also the intensities of the reflectors are affected^26^. Despite the poor quality of the pattern, it could be indexed as hydroxylapatite (Fig. 4g), with hexagonal-low (6/m) crystal symmetry, space group 176, which again was confirmed by the dynamic simulation shown in Fig. 4h. The quantitative EDX results with higher Ca/P-ratios as compared to the calcified plaque are in support of this finding, where minor fluorine end-member substitutions have taken place together with metal cationic substitutions of Na^+^ and Mg^2+^ and possibly SiO_4_^2−^ anionic complexes, quite analogous to the substitutions in the mineralization plaque. Both Calcium Phosphates, trigonal whitlockite and hexagonal hydroxylapatite, are closely related. The symmetry of the trigonal crystal system’s point lattice has reduced symmetry in comparison to the hexagonal point lattice due to the presence of additional lattice points at the centred lattice positions.

**Figure 5:**
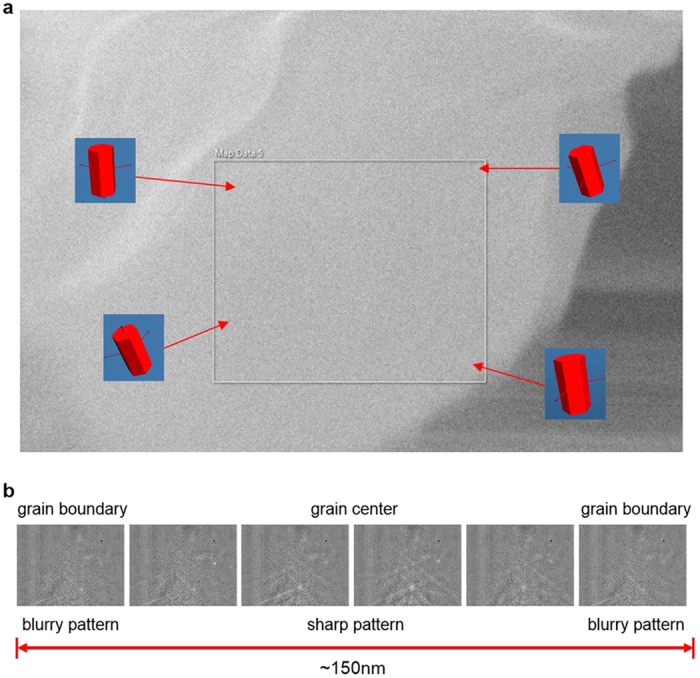
Change in orientation in the bone sample. Change in Kikuchipattern over a 760 × 620 nm area in the bone sample and the orientation of the unit cell, showing that the crystal orientation essentially stays the same within a few degrees (mean average deviation in the orientation determination due to low pattern quality few degrees). The bottom left pattern showed some distortion due to sample charging close to the cleavage edge and therefore also indexing was less accurate (**a**). The change in pattern intensity when moving the electron beam along a vertical line of 150 nm in steps of about 25 nm (**b**).

The Kikuchi pattern (TKD = transmission Kikuchi diffraction) taken from different points on the bone sample over an area of 760 × 620 nm did not show any significant change in crystal orientation, as can be seen in Fig. 5a. Two (100) planes are oriented parallel to the platelet surface, while the c-axis lies in the platelet plane, forming a sharp fibre texture. This is in good agreement with the work by Weiner *et al.*^27^ as well as the electron tomography observations made by Landis *et al.*^28^,^29^, who morphologically observed that the crystallographic c-axes of platelets are generally aligned parallel to one another and to the longitudinal collagen fibre. They furthermore describe platelets to be composed of fused smaller crystals, in each of which the c-axes are oriented parallel to the collagen fibre and (100) planes parallel to each other, forming coplanar larger units. The band contrast and sharpness of the pattern varied considerably over the mapped array, showing alternating sharpness when moving along a line, which is most likely due to smaller grains, i.e., mesocrystal character, showing rather sharp pattern in the centre which get blurry towards the grain boundaries (Fig. 5b). Judging from this, the grain size is roughly in the range of 120–150 nm.

## Discussion

In conclusion, spherical calcified particles occur isolated^5^, in the vicinity of larger calcific lesions, but also embedded within the lesions. While spherical particles are highly crystalline^5^, larger arterial plaques show colloidal nanograin structure. Nevertheless, electron backscatter diffraction pattern were acquired from regions where no obvious spheres were embedded, indicating polycrystalline nature of the lesions. The spherical particles exhibit concentric layers associated with degree of hydration and carbonatation, which is definitely an important fact in the search for the growth mechanisms and primal nucleation site. The distribution of hydrated and organic content in spherical particles is highly systematic on the nano-level, whereas in larger lesions distribution is rather inhomogenous on the micro scale, well in line with the different degree in crystallinity, possibly indicating differences in crystallization kinetics.

Basically, there are two possible formation routes for the concentric laminations observed in the spherical particles: The first would be ion-by-ion growth with layers possibly attributed to dietary growth rings. The other route would be via solidification of an amorphous precursor, where diffusion-limited exclusion of entrapped macromolecules can explain the concentric laminations. In this scenario, a fluidic mineral precursor can form some sort of mineral ‘cement’ that can then coalesce into a continuous mineral coating, possibly explaining also the morphology observed in the larger lesions^21^.

The resemblance that the plaque bears to bone is obvious in terms of both chemistry and crystal structure, as reported already by numerous studies^3^,^5^,^10^,^15^, where different calcium phosphate crystal structures have been reported of in conjunction with either structure. Also the variation in chemistry in the present study indicates that the presence of more than one calcium phosphate mineral structure is possible in both bone and plaque, even though only one phase was detected in both cases. Despite the similarities, the highly oriented directional growth of bone could not be observed in calcified lesions, but rather isotropic nature is prevalent, while, in turn, no spheres have been reported to be present at any stage of bone formation.

In focus of medical treatment of the coronary ischemic heart disease (CIHD) is the decline of coronary vessel narrowing progression. Herein, plaques are the pivotal element for the development of coronary stenosis, reduced blood flow and subsequent myocardial ischemia or infarction. Although the compact calcified plaque material is embedded in a cover of fibrocytes (see Fig. 1a), the architecture and composition of plaques should come in focus of interest for the establishment of new therapeutic tools. At present, patients suffering from CIHD are treated by lowering and controlling the lipid composition in blood, stenting of coronary arteries or, last but not at least, by the aortocoronary bypass surgical procedure. Better understanding of plaque composition as performed in our study could help to introduce new strategies for hydroxylapatite/whitelockite- dissolution inside the plaques, so that coronary lumina get free from the arteriosclerotic narrowing of the vessel. Studies on the manipulation and modification of Whitelockite biocompatibility are known from the fields of bioceramics, bone implants and osteology^30^,^31^ as well as dentistry^32^. A method for dissolution of the Whitelockite crystal structure could provide a new tool for CIHD medical treatment in a similar manner as mentioned in case of cholesterol monohydrate crystals before^33^.

## Methods

**Figure 6:**
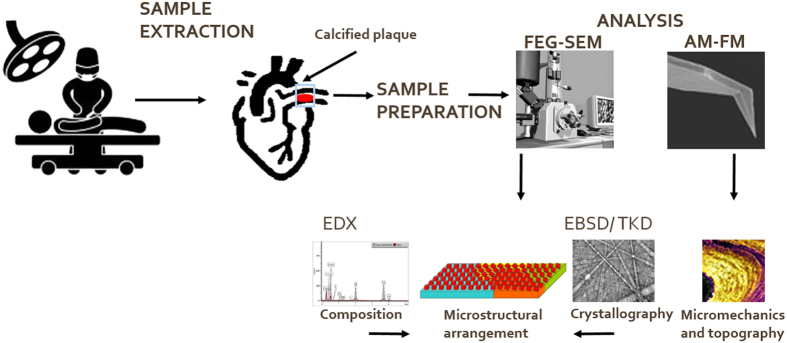
Schematic sketch of the major steps involved in the study. The images of the FEG–SEM, AM–FM tip, and AM–FM micromechanics and topography scan are image courtesy of Oxford Instuments plc. The illustration of the heart surgery is image courtesy of leremy/Depositphotos.com.

Figure 6 shows a schematic sketch illustrating the major steps of the study.

EBSD and TKD, enable the crystallographic analysis of material at nanometer resolution using the SEM, avoiding some of the limitations of the TEM, such as a rather limited field of view, a narrow diffraction angle requiring rotation of the sample in order to achieve different diffraction conditions as well as high beam energy and hence possible beam damage. Furthermore, individual EBSD patterns contain more detailed, multiple zone-axis crystallographic information than single selected area electron diffraction pattern, where information is obtained from a single zone axis only. Complementary, AM–FM viscoelastic mapping was used in this study, reaching a comparable nanoscale resolution. The local chemistry was determined using EDX, which is limited to lower spatial resolution magnitudes. While accurate quantification of calcium phosphates is rather challenging using EDX, the use of Ca/P-ratios is commonplace. Determination of the Ca/P-ratio, i.e., the ratio of the major constituents of hydroxylapatite and other calcium phosphates, narrows down the potential phases during the phase identification process.

### Samples

Institutional Review Board approval was obtained and supported by the scientific council. The experimental protocols were approved by the ethics committee of The Philipp University of Marburg – Department of Medical Studies (registration: AZ 131/12; MR 01.2013) and have therefore been performed in accordance with the ethical standards laid down in the 1964 Declaration of Helsinki and its later amendments. The work presented here was carried out on *ex-vivo* calcified coronary artery specimens and a bovine trabecular bone sample. The human atherosclerotic tissue samples were obtained from pathological examination while the bovine bone sample was received from a common slaughterhouse. All samples were fixed in 4% formalin for up to 48 hours. After fixation, the samples were subjected to a postfixation procedure with osmium tetroxide followed by dehydration through graded alcohol series (70%, 90%, 96% and 100% ethanol). The samples were defatted two times for 24 hours in 100% xylene and then placed in a mixture of xylene/Technovit 9100New (stabilized basic solution) as intermediate medium, at 4 °C for 24 hours. Subsequently, the samples were stored in the fridge for 48 h, placed in the pre-infiltration - and infiltration solution. During the embedding procedure, an aluminium block pre-cooled to −20 °C, with hole drills for the embedding capsules, was used. The infiltrated samples were positioned in the embedding containers and completely covered with the polymerization mixture. In order to remove air bubbles from the mixture, the samples were placed in a cooled desiccator (4 °C) under low vacuum for ca. 10 minutes. The embedding capsules were sealed air tight with polyethylene foil and a cap, and left to polymerize at −8 °C ≤ T ≤ −20 °C in the freezer. Afterwards, undecalcified thin sections were prepared using a heavy duty microtome. Prior to EBSD and AM–FM measurements, the blocks were ground and polished at various polishing sequences up to 1 μm with diamond suspension and a finish with colloidal silica solution. Subsequently, the samples were carbon coated in order to reduce charging artefacts.

### SEM analyses

A Zeiss Supra 55 VP FEG-SEM with a Gemini column was used, complemented by additional SEM micrographs of the artery inner wall and calcified area of deplastisized samples using a Leitz-ISI scanning electron microscope.

### EBSD, and TKD analyses

EBSD analyses were performed at 8–12 kV acceleration voltage and around 2–3 nA beam current (60 um aperture blend on the Zeiss Supra 55 VP field emission gun - scanning electron microscope), using an Oxford Instruments NordlysNano EBSD-detector. The AZtec software suite was used for acquisition and processing of the data. The rather low accelerating voltage in terms of EBSD analysis was chosen in order to minimize beam damage and charging at the same time. For the TKD measurements on bone, the accelerating voltage was increased to 25 kV, which proved to provide electrons with sufficient energy to transmit through the sample. For both EBSD and TKD, 4 × 4 pixel binning was applied, resulting in 336 × 256 pixel resolution. The TKD analyses were acquired on a fracture surface of the bone sample, where a platelet thin enough to be transmitted by ‘low energy’ electrons (25 keV) protruded.

### SEM-EDX analyses

EDX spectra were acquired at 8 kV while mappings were acquired at 5 kV using an Oxford Instruments XMax 150 SDD detector in combination with the AZtec software suite.

### AM–FM analysis

Viscoelastic mapping (AM–FM) was performed with a Cypher™ S atomic force microscope from Asylum Research, which combines conventional tapping via amplitude modulation (AM) for topography mapping with high sensitivity frequency modulation (FM), allowing for quantitative nanomechanical stiffness or Young’s modulus mapping. Quantitative mapping requires the use of a reference standard material for calibration. For this purpose, a polycarbonate sample with a Young’s modulus of 2.6 GPa was used.

### Statistical analysis

Distribution of data were tested with the Kolmogorov- Smirnov- test. Further data analysis were provided by the nonparametric Wilcoxon–Mann–Whitney test.

## Additional Information

**How to cite this article**: Curtze, S. C. *et al.* Step down Vascular Calcification Analysis using State-of-the-Art Nanoanalysis Techniques. *Sci. Rep.* **6**, 23285; doi: 10.1038/srep23285 (2016).

## Figures and Tables

**Table 1:** Chemical composition comparison of the vascular calcification lesions and bovine bone.

Spectrum Label	O	F	Na	Mg	Si	P	S	Ca	Ca/P-ratio
Aorta calcification	64.6	0.4	0.6	0.6	0.1	13.5	–	20.2	<1.5
Standard deviation (n = 6)	0.77	0.2	0.05	0.03	0.95	0.18	0.02	0.56	0.05
Bone	63.4	0.3	0.5	0.5	2.5	12.7	0.1	20.0	1.6
Standard deviation (n = 6)	0.77	0.13	0.11	0.10	1.84	0.48	0.08	1.48	0.12

Spectra were normalized over the Ca-Kα Peak. Values are given in at %. Carbon was excluded from the quantitative results as deconvolution element due to its presence as coating element, contaminant from hydrocarbonates in the microscope chamber, as well as organic phase, while nitrogen was excluded due to its association with collagen. The standard deviation from six measurements is shown in each case.
